# Improving a novel quantitative randomized response method using auxiliary variable information

**DOI:** 10.1016/j.heliyon.2024.e40367

**Published:** 2024-11-13

**Authors:** Hamed Salemian, Eisa Mahmoudi, Osama Abdulaziz Alamri, Javid Shabbir

**Affiliations:** a Department of Statistics, Yazd University, Yazd, Iran; b Department of Statistics, University of Tabuk, Tabuk, Saudi Arabia; c Department of Statistics, University of Wah, Wah Cantt, Pakistan

**Keywords:** Randomized response, Sensitive variable, Non-response, Auxiliary variable, Ratio estimator, Product estimator

## Abstract

In the majority of sample surveys, the variable of interest might have sensitive characteristics. Since we frequently get unrealistic responses from respondents during interviews as employing the direct method is not seen logical in these situations. To tackle this difficulty, the randomized response technique is good substitute for the direct method. The respondent is reassured that there would not be any issues will your response when using the randomized response technique as it protects privacy and secrets. In this paper, we propose a novel quantitative three-stage randomized response technique. Using simulation with R software, we prove that the results obtained from the proposed method are ideal. We suggest ratio and product type estimators by using the auxiliary information to enhance the efficiency of estimators. It is demonstrated that these estimators are superior for the conventional ones in special situations and in applied task, the use of auxiliary variable improves the estimation of the average cheating of Shahid Chamran university of Ahvaz students. The main objective is to increase the average sensitive reaction by utilizing the auxiliary variable.

## 1 Introduction

In most survey sampling, the topics that are the target of the research are considered sensitive topics. Some of the sensitive topics include smoking, not following driving rules, cheating in exams, income, abortion, paying taxes, embezzlement, and gambling. Using the direct method in sampling sensitive issues is not an answer because if we use the direct method in sampling, we will encounter non-response or dishonest answers, which will cause bias and as a result, unreasonable estimates for the desired unknown parameters. To solve this problem, we use the randomized response method, which maintains confidentiality and the respondent's secret is not revealed, and is a suitable alternative to the direct method. This method eliminates the apprehension and worry of the responder that happens if the direct method is used and assures the responder that cooperation in this research will not have any consequences or problems for them and estimates with higher accuracy and reliability for unknown parameters results.

Randomized response methods are of two types. The first type is called qualitative randomized response methods and the second type is called quantitative randomized response methods. When the answers to the questions are two-mode, we use the qualitative randomized response method. In such a case, the sensitive variable has only two values. The qualitative randomized response method is widely used in medical and social science research. Recent researches in this field include: Singh and Tarray [26], Singh and Gorey [23], Abid et al. [1], Shah et al. [22], Narjis and Shabbir [17] and Aboalkhair et al. [2].

In the rest of this section, we describe a historical background of the quantitative randomized response method.

Warner [28] presented a new linear randomized response method. Eichhorn and Hayre [7] introduced a multiplicative randomized response method. Gupta et al. [11] estimated the level of sensitivity of personal interview survey questions using the randomized response method. Bouza [5] estimated the mean of a quantitatively sensitive variable using a randomized response method under ranked set sampling. Gupta et al. [12] estimated the mean and sensitivity level of a sensitive question using a two-stage randomized response method. Arnab and Singh [3] introduced a new estimator using the successive sampling method for the mean of the sensitive variable. Hussain and Al-Zahrani [14] estimated the level of sensitivity and mean using the randomized response technique. Narjis and Shabbir [17] proposed a new randomized response method and showed that the proposed method is superior to the method of Gjestvang and Singh [8] under SRSWR and successive sampling. Tiwari et al. [27] estimated the mean sensitive variable of the population under the influence of measurement error using the randomized response method. Azeem et al. [4] introduced a new randomized response method based on the randomized response method of Narjis and Shabbir [17] and showed that the proposed method is better than Narjis and Shabbir's method in estimating the mean of the sensitive variable.

In the following, we will review important recent researches in the field of improving the estimation of the mean of the sensitive variable using auxiliary variable information. Sousa et al. [25] proposed a ratio estimator for the mean of the sensitive variable using auxiliary information. Gupta et al. [13] designed a regression estimator for the mean of the sensitive variable, showed that the regression estimator is better than the ratio estimator and the traditional mean estimator (without using auxiliary information). Sousa et al. [24] obtained ratio and regression estimators using the stratified random sampling method and based on the randomized response method. Tarray and Singh [26] introduced new ratio and regression estimators, which are better than ratio and regression estimators of Sousa et al. [25] and Gupta et al. [13]. Hussain and Arshad [15] improved the estimation of the mean of the sensitive variable using an optional randomized response method and with the help of non-sensitive auxiliary information. Khalil et al. [16] were able to propose an improved estimate for the mean of the sensitive variable by using an optional randomized response method when there is measurement error. Grover and Kaur [10] introduced an improved estimator of the regression type based on the robust regression method using the information of two auxiliary variables. Zahid et al. [29] proposed a new class of sensitive variable mean estimators in the presence of measurement error and non-response under stratified random sampling. Patidar and Singh [19] proposed a new class of population mean estimators under a randomized response method and showed in an experimental study that the proposed class is better than the Grover and Kaur [9] estimator. Qureshi et al. [20] proposed two Ln type estimators to improve the estimation of the mean of the sensitive variable by using auxiliary information. Onyango et al. [18] proposed a generalized class of estimators for the mean of the sensitive variable using a three-stage randomized response method in the presence of measurement error and non-response.

The rest of the paper is as follows:

In Section 2, the suggested method is presented, and using it, an unbiased estimate for the mean of the sensitive variable is introduced and an unbiased estimate for its variance is presented. In order to analyze the theoretical findings, in Section 3, we will present the simulation results of the proposed method. In Section 4, using the auxiliary information, the ratio and product estimation of the sensitive variable average for the suggested method are presented. In Section 5, the superiority of the introduced ratio and product estimation compared to the suggested traditional method is expressed in the form of two theorems. Finally, in an experimental study, ratio and product estimates will be calculated using auxiliary information.

## 2 Suggested randomized response method

To increase the confidentiality of sensitive question answers, a new quantitative randomized response method is suggested using a three-stage random experiment. Using this method, an estimate for the mean of the sensitive variable along with its variance is provided. Suppose *Y* is a sensitive variable with mean and standard deviation $\mu_{y}$ and $\sigma_{y}$, respectively and *S* is a variable with known distribution which is independent of *Y*. We assume that the domain of both variables is the interval (a,b). The randomized response method is that we ask each person to first perform a three-state test (*T*) as follows:

$$T=\left\{ \begin{array}{cc} 1, & p_{1},\\ 2, & p_{2},\\ 3, & p_{3}, \end{array}\right.$$

where $p_{3}=1-p_{1}-p_{2}$. If the first state (T=1) occurs, the response (*S*), if the second state (T=2) occurs, the sensitive response (*Y*) and otherwise the sensitive response (b−Y) reported in the answer sheet, therefore, the randomized response variable (*Z*) is defined as follows:

$$Z=\left\{ \begin{array}{cc} S, & p_{1},\\ Y, & p_{2},\\ b-Y, & p_{3}, \end{array}\right.$$

where *b* is fixed value whose value's is determined according to the investigated problem and $\sum_{i=1}^{3}p_{i}=1$. The expectation of the randomized response variable *Z* under the randomized technique is as follows:

$$E_{R}(Z|Y)=p_{1}\mu_{s}+(\;p_{2}-\;p_{3})Y+p_{3}b.$$

Therefore, the expectation of *Z* based on the conditional expectation rule is obtained as follows:

$$E(Z)=E_{Y}(E_{R}(Z|Y))=p_{1}\mu_{s}+p_{3}b+(p_{2}-p_{3})\mu_{y}.$$

An estimator for the mean of quantitatively sensitive variable *Y*, based on a random sample $Z_{1},\ldots ,Z_{n}$, is defined as

(1) $${\overset{ˆ}{\mu }}_{y}=\frac{\overset{¯}{Z}-p_{1}\mu_{s}-p_{3}b}{p_{2}-p_{3}},$$

where $\overset{¯}{Z}$ is the sample mean of randomized responses and $p_{2}\neq p_{3}$. Note that $E({\overset{ˆ}{\mu }}_{y})=\mu_{y}$. The variance of the estimator of the mean of the sensitive variable is equal to

(2) $$V({\overset{ˆ}{\mu }}_{y})=\frac{V(\overset{¯}{Z})}{{\left(p_{2}-p_{3}\right)}^{2}}=\frac{V(Z)}{n{\left(p_{2}-p_{3}\right)}^{2}},$$

where the variance of *Z* is obtained according to the following equation:

(3) $$V(Z)=V_{Y}(E_{R}(Z|Y))+E_{Y}(V_{R}(Z|Y)).$$

The components of V(Z) are obtained as follows:

(4) $$V_{Y}(E_{R}(Z|Y))=V_{Y}(p_{1}\mu_{s}+\;p_{3}b+\;(p_{2}-p_{3})Y)={\left(p_{2}-p_{3}\right)}^{2}\sigma_{y}^{2},$$

and

(5) $$V_{R}(Z|Y)=E_{R}(Z^{2}|Y)-{\left(E_{R}(Z|Y)\right)}^{2}=p_{1}E(S^{2})+p_{2}Y^{2}+p_{3}{\left(b-Y\right)}^{2}-{\left(p_{1}\mu_{s}+\;p_{3}b+(p_{2}-p_{3})Y\right)}^{2}=p_{1}(\sigma_{s}^{2}+\mu_{s}^{2})+p_{2}Y^{2}+p_{3}(b^{2}+Y^{2}-2bY)-{\left(p_{1}\mu_{s}+\;p_{3}b\right)}^{2}-{\left(p_{2}-p_{3}\right)}^{2}Y^{2}-2(p_{1}\mu_{s}+\;p_{3}b)(p_{2}-p_{3})Y=\left[p_{2}+p_{3}-{\left(p_{2}-p_{3}\right)}^{2}\right]Y^{2}-2[p_{3}b+(p_{1}\mu_{s}+\;p_{3}b)(p_{2}-p_{3})]Y+p_{1}(\sigma_{s}^{2}+\mu_{s}^{2})+p_{3}b^{2}-{\left(p_{1}\mu_{s}+p_{3}b\right)}^{2}.$$

In the above equations R is the index represents the variance under the Randomized technique. Therefore, by substituting Eqs. (4) and (5) in to (3) one can obtain V(Z) as follows:

$$V(Z)={\left(p_{2}-p_{3}\right)}^{2}\sigma_{y}^{2}+(p_{2}+p_{3}-{\left(p_{2}-p_{3}\right)}^{2})(\sigma_{y}^{2}+\mu_{y}^{2})-2[p_{3}b+(p_{1}\mu_{s}+p_{3}b)(p_{2}-p_{3})]\mu_{y}+p_{1}(\sigma_{s}^{2}+\mu_{s}^{2})+p_{3}b^{2}-{\left(p_{1}\mu_{s}+p_{3}b\right)}^{2}.$$

By substituting $s_{z}^{2}$ instead of V(Z) in Eq. (2), we have

$$\overset{ˆ}{V}({\overset{ˆ}{\mu }}_{y})=\frac{s_{z}^{2}}{n{\left(p_{2}-p_{3}\right)}^{2}}.$$

Under a simple random sampling without replacement (SRSWOR) from a finite population of size *N*, the variance of the suggested estimator is given by

(6) $$V({\overset{ˆ}{\mu }}_{y})=(\frac{1}{n}-\frac{1}{N})\frac{S_{z}^{2}}{{\left(p_{2}-p_{3}\right)}^{2}},$$

where $S_{z}^{2}=\frac{\sum_{i=1}^{N}{\left(Z_{i}-\mu_{z}\right)}^{2}}{N-1}=\frac{N}{N-1}V(Z)$.

## 3 Simulation study

**Figure 1:**
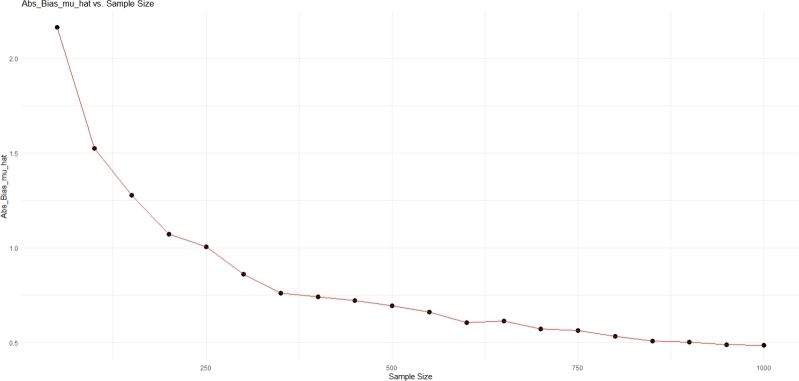
Plot of $\left|\overset{ˆ}{Bias}({\overset{ˆ}{\mu }}_{y})\right|$ versus sample size for *μ*_*y*_ = 5.

**Figure 2:**
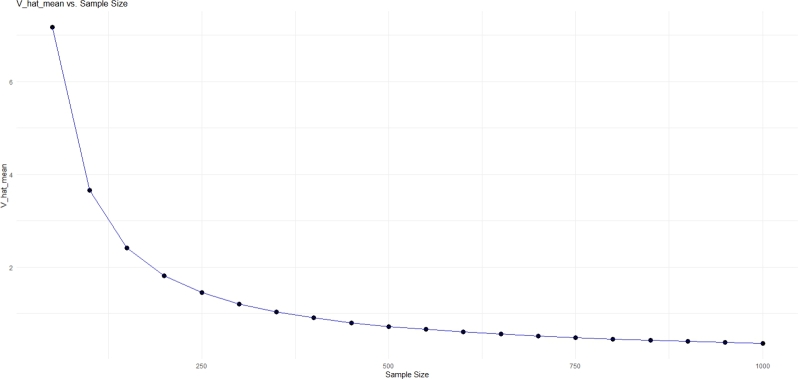
Plot of $\overset{ˆ}{V}({\overset{ˆ}{\mu }}_{y})$ versus sample size for *μ*_*y*_ = 5 and *σ*_*y*_ = 5.

In this section, we give some simulation using R to clarify performance of the mean estimator and variance estimator of the sensitive variable. In this study, the Monte Carlo simulation method is used and the simulation results for the values $\mu_{y}=(5,10,20)$, $\sigma_{y}=(5,10,15)$ and the sample size n=(50,100,300,500) are given in Table 1 and Figure 1, Figure 2.

**Table 1:** Simulation results for different values *μ*_*y*_, *σ*_*y*_ and sample size.

Sample Size	*μ*_*y*_	*σ*_*y*_	$\overset{ˆ}{E}({\overset{ˆ}{\mu }}_{y})$	$\left|\overset{ˆ}{Bias}({\overset{ˆ}{\mu }}_{y})\right|$	$\overset{ˆ}{V}({\overset{ˆ}{\mu }}_{y})$
50	5	5	4.974594	2.1437927	7.2773002
100			5.038292	1.5924498	3.6066209
300			4.966614	0.8470644	1.2052152
500			4.951079	0.6678687	0.7289187

50	5	10	5.118656	4.0393636	25.3559771
100			5.116315	2.7219008	12.6528374
300			5.082558	1.5982148	4.2096314
500			4.991821	1.3017087	2.5167658

50	5	15	5.232149	6.0706548	54.9221570
100			5.116099	4.3911995	27.7373768
300			4.967919	2.4317153	9.2109060
500			4.972208	1.8589176	5.5194453

50	10	5	9.894694	2.8978898	12.9822982
100			10.104967	2.0898540	6.5066017
300			9.941536	1.1767628	2.1699279
500			9.915121	0.9368027	1.2941578

50	10	10	9.890644	4.4002980	30.6616997
100			10.098513	3.2300713	15.3663197
300			10.033642	1.7807990	5.1667757
500			10.043700	1.4037250	3.1002312

50	10	15	10.134307	6.2309593	60.9590363
100			9.807570	4.3277583	30.4768607
300			9.970774	2.6459909	10.1581227
500			10.058894	1.9007782	6.0796952

50	20	5	19.918673	6.1956504	57.2850030
100			19.952083	4.4186969	28.6590462
300			19.952385	2.4300146	9.5771008
500			19.983721	1.9282304	5.7321894

50	20	10	19.810195	6.9224898	74.9957744
100			20.046220	5.0324598	37.6262515
300			20.008646	2.8247780	12.6253760
500			20.013465	2.1621022	7.5544555

50	20	15	19.030104	8.4172359	106.0648532
100			19.989553	5.7420335	52.7411768
300			20.105200	3.3786169	17.5006422
500			19.946709	2.4927743	10.5256157

Note that $\overset{ˆ}{E}({\overset{ˆ}{\mu }}_{y})$ and $\overset{ˆ}{V}({\overset{ˆ}{\mu }}_{y})$ are the estimated values of $E({\overset{ˆ}{\mu }}_{y}),V({\overset{ˆ}{\mu }}_{y})$ and $\left|\overset{ˆ}{Bias}({\overset{ˆ}{\mu }}_{y})|=|\overset{ˆ}{E}({\overset{ˆ}{\mu }}_{y})-E({\overset{ˆ}{\mu }}_{y})\right|$.

Based on Table 1 and Figure 1, Figure 2, it can be seen that $\left|\overset{ˆ}{Bias}({\overset{ˆ}{\mu }}_{y})\right|$ decreases for different values of parameter $\mu_{y}$ and $\sigma_{y}$ with increasing sample size and becomes close to zero, so it can be concluded that the mean estimator of the sensitive variable is almost unbiased and works well. The value of $\overset{ˆ}{V}({\overset{ˆ}{\mu }}_{y})$ decreases as the sample size increases, so we will see decreasing behavior in error and dispersion, and it indicates the good performance of the variance estimator. Based on these results, the accuracy of the theoretical results is confirmed.

## 4 Introducing ratio and product estimators using auxiliary information

Assuming $\mu_{x}$ is known, to estimate $\mu_{y}$ using the auxiliary variable *X*, based on Cochran [6], the ratio estimator is given by

$${\overset{ˆ}{\mu }}_{R}=\frac{{\overset{ˆ}{\mu }}_{y}}{\overset{¯}{x}}\mu_{x},$$

and based on Robson [21], the product estimator is given by

$${\overset{ˆ}{\mu }}_{P}=\frac{{\overset{ˆ}{\mu }}_{y}}{\mu_{x}}\overset{¯}{x}.$$

Using (1), the estimator ${\overset{ˆ}{\mu }}_{y}$ can be expressed as

(7) $${\overset{ˆ}{\mu }}_{y}=a\overset{¯}{z}+b,$$

where $a=\frac{1}{p_{2}-p_{3}}$, $b=-\frac{p_{1}\mu_{s}+p_{3}b}{p_{2}-p_{3}}$, and $\overset{¯}{z}$ is the sample mean of randomized responses. Since $\overset{¯}{x}$ and ${\overset{ˆ}{\mu }}_{y}$ are unbiased estimators for $\mu_{x}$ and $\mu_{y}$, the following models can be defined as

(8) $${\overset{ˆ}{\mu }}_{y}=\mu_{y}+\varepsilon_{0},$$

(9) $$\overset{¯}{x}=\mu_{x}+\varepsilon_{1}.$$

According to models (8) and (9), the ratio estimator of ${\overset{ˆ}{\mu }}_{R}$ can be rewritten as

$${\overset{ˆ}{\mu }}_{R}=\frac{\mu_{y}+\varepsilon_{0}}{\mu_{x}+\varepsilon_{1}}\mu_{x}=\mu_{y}(1+\frac{\varepsilon_{0}}{\mu_{y}}){\left(1+\frac{\varepsilon_{1}}{\mu_{x}}\right)}^{-1}.$$

Thus, we have

(10) $${\overset{ˆ}{\mu }}_{R}≅\mu_{y}(1+\frac{\varepsilon_{0}}{\mu_{y}}-\frac{\varepsilon_{1}}{\mu_{x}}+\frac{\varepsilon_{1}^{2}}{\mu_{x}^{2}}-\frac{\varepsilon_{0}\varepsilon_{1}}{\mu_{x}\mu_{y}}).$$

The relation (10) can be simplified as

(11) $${\overset{ˆ}{\mu }}_{R}-\mu_{y}≅\mu_{y}(\frac{\varepsilon_{1}^{2}}{\mu_{x}^{2}}-\frac{\varepsilon_{0}\varepsilon_{1}}{\mu_{x}\mu_{y}})=\mu_{y}(\frac{V(\varepsilon_{1})}{\mu_{x}^{2}}-\frac{Cov(\varepsilon_{0},\varepsilon_{1})}{\mu_{x}\mu_{y}}).$$

Taking expectation from two sides of expression (11), the bias of ${\overset{ˆ}{\mu }}_{R}$ is obtained as follows:

$$\mathrm{Bias}({\overset{ˆ}{\mu }}_{R})≅\mu_{y}(\frac{V(\overset{¯}{x})}{\mu_{x}^{2}}-\frac{Cov({\overset{ˆ}{\mu }}_{y},\overset{¯}{x})}{\mu_{x}\mu_{y}}).$$

By substituting Eq. (7) in to term $Cov({\overset{ˆ}{\mu }}_{y},\overset{¯}{x})$, we have

$$\mathrm{Bias}({\overset{ˆ}{\mu }}_{R})≅\mu_{y}(\frac{V(\overset{¯}{x})}{\mu_{x}^{2}}-\frac{Cov(a\overset{¯}{z}+b,\overset{¯}{x})}{\mu_{x}\mu_{y}})≅\mu_{y}(\frac{V(\overset{¯}{x})}{\mu_{x}^{2}}-\frac{aCov(\overset{¯}{z},\overset{¯}{x})}{\mu_{x}\mu_{y}}).$$

Under SRSWOR, we have

$$V(\overset{¯}{x})=(\frac{1}{n}-\frac{1}{N})S_{x}^{2},\;\;Cov(\overset{¯}{x},\overset{¯}{z})=(\frac{1}{n}-\frac{1}{N})S_{xz}.$$

According to models (8) and (9), the product estimator of ${\overset{ˆ}{\mu }}_{P}$ is rewritten as

$${\overset{ˆ}{\mu }}_{P}≅\mu_{y}(1+\frac{\varepsilon_{0}}{\mu_{y}})\mu_{x}(1+\frac{\varepsilon_{1}}{\mu_{x}})\frac{1}{\mu_{x}}.$$

Thus,

$${\overset{ˆ}{\mu }}_{P}-\mu_{y}≅\mu_{y}(\frac{\varepsilon_{1}}{\mu_{x}}+\frac{\varepsilon_{0}}{\mu_{y}}+\frac{\varepsilon_{0}\varepsilon_{1}}{\mu_{y}\mu_{x}}).$$

By ignoring first-order sentences, we have

(12) $${\overset{ˆ}{\mu }}_{P}-\mu_{y}≅\mu_{y}(\frac{Cov(\varepsilon_{0},\varepsilon_{1})}{\mu_{x}\mu_{y}}).$$

Taking expectation from two sides of Expression (12), the bias of ${\overset{ˆ}{\mu }}_{P}$ is obtained by

$$\mathrm{Bias}({\overset{ˆ}{\mu }}_{P})≅\mu_{y}(\frac{Cov({\overset{ˆ}{\mu }}_{y},\overset{¯}{x})}{\mu_{x}\mu_{y}}).$$

Thus, according to relation (7), one can obtain:

$$\mathrm{Bias}({\overset{ˆ}{\mu }}_{P})≅\mu_{y}(\frac{aCov(\overset{¯}{z},\overset{¯}{x})}{\mu_{x}\mu_{y}}).$$

Under SRSWOR, we have

$$\mathrm{Bias}({\overset{ˆ}{\mu }}_{p})≅(\frac{1}{n}-\frac{1}{N})\frac{aS_{xz}}{\mu_{x}}\;.$$

With the first-order approximation, the MSEs of the estimator ${\overset{ˆ}{\mu }}_{R}$ and ${\overset{ˆ}{\mu }}_{P}$ are obtained as follows:

$$\mathrm{MSE}({\overset{ˆ}{\mu }}_{R})≅\mu_{y}^{2}\left[\frac{V({\overset{ˆ}{\mu }}_{y})}{\mu_{y}^{2}}+\frac{V(\overset{¯}{x})}{\mu_{x}^{2}}-2\frac{Cov({\overset{ˆ}{\mu }}_{y},\overset{¯}{x})}{\mu_{x}\mu_{y}}\right].$$

According to (7), we have

$$\mathrm{MSE}({\overset{ˆ}{\mu }}_{R})≅\mu_{y}^{2}\left[\frac{a^{2}V(\overset{¯}{z})}{\mu_{y}^{2}}+\frac{V(\overset{¯}{x})}{\mu_{x}^{2}}-\frac{2aCov(\overset{¯}{x},\overset{¯}{z})}{\mu_{x}\mu_{y}}\right].$$

Thus, under SRSWOR, we have

$$\mathrm{MSE}({\overset{ˆ}{\mu }}_{R})≅(\frac{1}{n}-\frac{1}{N})\mu_{y}^{2}\left[\frac{a^{2}S_{z}^{2}}{\mu_{y}^{2}}+\frac{S_{x}^{2}}{\mu_{x}^{2}}-\frac{2aS_{xz}}{\mu_{x}\mu_{y}}\right].$$

As a result, by inserting R, we have

$$\mathrm{MSE}({\overset{ˆ}{\mu }}_{R})≅(\frac{1}{n}-\frac{1}{N})\left[a^{2}S_{z}^{2}+R^{2}S_{x}^{2}-2aRS_{xz}\right],$$

where $R=\frac{\mu_{y}}{\mu_{x}}$. An estimate for the MSE using a SRSWOR is given by

(13) $$\overset{ˆ}{\mathrm{MSE}}({\overset{ˆ}{\mu }}_{R})≅(\frac{1}{n}-\frac{1}{N})\left[r^{2}s_{x}^{2}+a^{2}s_{z}^{2}-2ars_{xz}\right].$$

It is introduced that $r=\frac{{\overset{ˆ}{\mu }}_{y}}{\overset{¯}{x}},s_{x}^{2},s_{z}^{2}$ and $s_{xz}$ are the sample variance of auxiliary variable, the randomized responses and the sample covariance between *X* and *Z*, respectively. These values are defined by

$$s_{x}^{2}=\frac{\sum_{i=1}^{n}{\left(x_{i}-\overset{¯}{x}\right)}^{2}}{n-1},s_{z}^{2}=\frac{\sum_{i=1}^{n}{\left(z_{i}-\overset{¯}{z}\right)}^{2}}{n-1},s_{xz}=\frac{\sum_{i=1}^{n}(x_{i}-\overset{¯}{x})(z_{i}-\overset{¯}{z})}{n-1},$$

with a similar trend as for ${\overset{ˆ}{\mu }}_{R}$, we have for ${\overset{ˆ}{\mu }}_{P}$:

$${\overset{ˆ}{\mu }}_{P}-\mu_{y}≅\mu_{y}(\frac{\varepsilon_{1}}{\mu_{x}}+\frac{\varepsilon_{0}}{\mu_{y}}).\mathrm{MSE}({\overset{ˆ}{\mu }}_{P})≅\mu_{y}^{2}(\frac{V({\overset{ˆ}{\mu }}_{y})}{\mu_{y}^{2}}+\frac{V(\overset{¯}{x})}{\mu_{x}^{2}}+2\frac{Cov(\overset{¯}{x},{\overset{ˆ}{\mu }}_{y})}{\mu_{y}\mu_{x}}).$$

According to Eq. (7), we have

$$\mathrm{MSE}({\overset{ˆ}{\mu }}_{P})≅\mu_{y}^{2}\left[\frac{a^{2}V(\overset{¯}{z})}{\mu_{y}^{2}}+\frac{V(\overset{¯}{x})}{\mu_{x}^{2}}+\frac{2aCov(\overset{¯}{x},\overset{¯}{z})}{\mu_{x}\mu_{y}}\right].$$

Under SRSWOR, one can obtain

$$\mathrm{MSE}({\overset{ˆ}{\mu }}_{P})≅(\frac{1}{n}-\frac{1}{N})\mu_{y}^{2}\left[\frac{a^{2}S_{z}^{2}}{\mu_{y}^{2}}+\frac{S_{x}^{2}}{\mu_{x}^{2}}+\frac{2aS_{xz}}{\mu_{x}\mu_{y}}\right].$$

As a result, by putting $R=\frac{\mu_{y}}{\mu_{x}}$, we have

(14) $$\mathrm{MSE}({\overset{ˆ}{\mu }}_{P})≅(\frac{1}{n}-\frac{1}{N})[a^{2}S_{z}^{2}+R^{2}S_{x}^{2}+2aRS_{xz}].$$

An estimate for the MSE in Eq. (14), using SRSWOR is given by

(15) $$\overset{ˆ}{\mathrm{MSE}}({\overset{ˆ}{\mu }}_{P})≅(\frac{1}{n}-\frac{1}{N})\left[r^{2}s_{x}^{2}+a^{2}s_{z}^{2}+2ars_{xz}\right].$$

## 5 Superiority of ratio and product estimators

In this section, the superiority of the ratio and product estimator compared to the suggested traditional method is expressed in the form of two propositions.

Proposition 1 *Under SRSWOR, the ratio estimator*${\overset{ˆ}{\mu }}_{R}=\frac{{\overset{ˆ}{\mu }}_{y}}{\overset{¯}{x}}\mu_{x}$*is better than the usual suggested estimator*${\overset{ˆ}{\mu }}_{y}$*if*

$$\rho_{xz}>\frac{\left(p_{2}-p_{3}\right)}{2}R\frac{S_{x}}{S_{z}},$$

*where*$R=\frac{\mu_{y}}{\mu_{x}}$*and*$\rho_{xz}$*is the correlation between auxiliary variable and randomized response.*

Proof The ratio estimator is better than the suggested estimator if

$$V({\overset{ˆ}{\mu }}_{y})>MSE({\overset{ˆ}{\mu }}_{R}),$$

hence

$$V({\overset{ˆ}{\mu }}_{y})-MSE({\overset{ˆ}{\mu }}_{R})>0,$$

thus, we have

(16) $$(\frac{1}{n}-\frac{1}{N})\frac{S_{z}^{2}}{{\left(p_{2}-p_{3}\right)}^{2}}-(\frac{1}{n}-\frac{1}{N})[a^{2}S_{z}^{2}+R^{2}S_{x}^{2}-2aRS_{xz}]>0.$$

By substituting $a=\frac{1}{p_{2}-p_{3}}$ and $R=\frac{\mu_{y}}{\mu_{x}}$ in to Eq. (16), we have

$$(\frac{1}{n}-\frac{1}{N})\frac{S_{z}^{2}}{{\left(p_{2}-p_{3}\right)}^{2}}-(\frac{1}{n}-\frac{1}{N})[\frac{S_{z}^{2}}{{\left(p_{2}-p_{3}\right)}^{2}}+\frac{\mu_{y}^{2}}{\mu_{x}^{2}}S_{x}^{2}-\frac{2}{\left(p_{2}-p_{3}\right)}\frac{\mu_{y}}{\mu_{x}}S_{xz}]>0,$$

then

$$-(\frac{1}{n}-\frac{1}{N})\frac{\mu_{y}^{2}}{\mu_{x}^{2}}S_{x}^{2}+(\frac{1}{n}-\frac{1}{N})\frac{2}{\left(p_{2}-p_{3}\right)}\frac{\mu_{y}}{\mu_{x}}S_{xz}>0.$$

By simple calculations, we have

(17) $$S_{xz}>\frac{\frac{\mu_{y}^{2}}{\mu_{x}^{2}}S_{x}^{2}}{\frac{2}{\left(p_{2}-p_{3}\right)}\frac{\mu_{y}}{\mu_{x}}}.$$

Dividing the both sides of Eq. (17) by $S_{x}S_{z}$, gives

$$\frac{S_{xz}}{S_{x}S_{z}}>\frac{\left(p_{2}-p_{3}\right)}{2}\frac{\mu_{y}}{\mu_{x}}\frac{S_{x}}{S_{z}},$$

which implify

$$\rho_{xz}>\frac{\left(p_{2}-p_{3}\right)}{2}R\frac{S_{x}}{S_{z}}\;.$$

The proof is completed. □

Proposition 2 *Under SRSWOR, the product estimator*${\overset{ˆ}{\mu }}_{P}=\frac{{\overset{ˆ}{\mu }}_{y}}{\mu_{x}}\overset{¯}{x}$*is better than the usual suggested estimator*${\overset{ˆ}{\mu }}_{y}$*if*

$$\rho_{xz}<-\frac{\left(p_{2}-p_{3}\right)}{2}\;R\;\frac{S_{x}}{S_{z}},$$

*where*$R=\frac{\mu_{y}}{\mu_{x}}$*and*$\rho_{xz}$*is the correlation between auxiliary variable and randomized response.*

Proof The product estimator is more efficient than the suggested estimator if

$$V({\overset{ˆ}{\mu }}_{y})>MSE({\overset{ˆ}{\mu }}_{P}),$$

then

$$V({\overset{ˆ}{\mu }}_{y})-MSE({\overset{ˆ}{\mu }}_{p})>0.$$

By inserting relations (6) and (14), in above Eq., we have

$$(\frac{1}{n}-\frac{1}{N})\frac{S_{z}^{2}}{{\left(p_{2}-p_{3}\right)}^{2}}-(\frac{1}{n}-\frac{1}{N})[a^{2}S_{z}^{2}+R^{2}S_{x}^{2}+2aRS_{xz}]>0.$$

Substituting the values of *a* and *R* and simplifying the sentences, gives

$$-(\frac{1}{n}-\frac{1}{N})\frac{\mu_{y}^{2}}{\mu_{x}^{2}}S_{x}^{2}-(\frac{1}{n}-\frac{1}{N})\frac{2}{\left(p_{2}-p_{3}\right)}\frac{\mu_{y}}{\mu_{x}}S_{xz}>0,$$

which implify to

$$-S_{xz}>\frac{\frac{\mu_{y}^{2}}{\mu_{x}^{2}}S_{x}^{2}}{\frac{2}{\left(p_{2}-p_{3}\right)}\frac{\mu_{y}}{\mu_{x}}}.$$

In the end, by dividing the sentences on both sides by $S_{x}S_{z}$, we have

$$\frac{S_{xz}}{S_{x}S_{z}}<-\frac{\left(p_{2}-p_{3}\right)}{2}\frac{\mu_{y}}{\mu_{x}}\frac{S_{x}}{S_{z}},$$

which results

$$\rho_{xz}<-\frac{\left(p_{2}-p_{3}\right)}{2}R\frac{S_{x}}{S_{z}}.$$

Thus, the proof is completed. □

## 6 Empirical study

Cheating students in exams every semester is one of the common problems that every university faces. The existence of such a problem can cause any student's academic failure and bring irreparable consequences, including probation and expulsion of the student. Knowing the average number of cheating students per semester for each university can lead to better decision-making and planning for the academic progress of students of that university. Because the question about cheating in exams is considered as one of the sensitive issues, therefore, it cannot be expected that the use of the direct method will bring good results. Because we face unanswered and dishonest or unrealistic answers. For this reason, we go to the randomized response method. The use of this method makes the responder complete the questionnaire without fear and apprehension of the possible consequences and increases the participation rate compared to the direct method. In this article, we estimate the average number of cheating in the exams using auxiliary information (GPA) for the students of Shahid Chamran University of Ahvaz in the first half of the academic year 2018-2019. The statistical population is undergraduate students of Shahid Chamran University of Ahvaz. A stratified random sampling method has been used. Each college is considered one stratum. Each student is requested to answer the questionnaire according to the instructions mentioned in it. The instructions are as follows: first, the responder is asked to randomly choose a card from the deck of cards with one of the numbers 0 to 9 written on it, and without anyone noticing the number on the card, put it to remember and then toss a coin twice away from the eyes of others. The desired coin is homogeneous and the probability of heads and tails is the same. If it is a lion both times by tossing a coin, enter only the number on the card in the randomized answer section of the answer sheet, which in this case is $p_{1}=0.25$ for the first case. If the line appears both times, enter only the number of cheating in the last semester in the randomized answer section of the answer sheet. For this case, $p_{2}=0.25$. If a lion and a line appear in the answer sheet in the randomized answer section, reduce the number of frauds from the number 9 and enter only the result. For this case, $p_{3}=0.5$.

The ratio and product estimates of the average number of cheating for the $h^{th}$ stratum (College) based on randomized responses is equal to

$${\overset{ˆ}{\mu }}_{Rh}=\frac{{\overset{ˆ}{\mu }}_{yh}}{{\overset{¯}{x}}_{h}}\mu_{xh},{\overset{ˆ}{\mu }}_{Ph}=\frac{{\overset{ˆ}{\mu }}_{yh}}{\mu_{xh}}{\overset{¯}{x}}_{h}.$$

The third and fourth columns of Table 4 show the ratio and product estimates of the average number of cheating students of each college of Shahid Chamran University of Ahvaz. The estimation of their variance is based on (13) and (15) in the fifth and sixth columns of Table 4, respectively. Ratio and product estimation of the average number of cheating and its variance for the population (university) obtain from the following Eqs:

(18) $${\overset{ˆ}{\mu }}_{Rst}=\sum_{h=1}^{L}W_{h}{\overset{ˆ}{\mu }}_{Rh},$$

(19) $$\overset{ˆ}{V}({\overset{ˆ}{\mu }}_{Rst})=\sum_{h=1}^{L}W_{h}^{2}\overset{ˆ}{V}({\overset{ˆ}{\mu }}_{Rh}),$$

(20) $${\overset{ˆ}{\mu }}_{Pst}=\sum_{h=1}^{L}W_{h}{\overset{ˆ}{\mu }}_{Ph},$$

(21) $$\overset{ˆ}{V}({\overset{ˆ}{\mu }}_{Pst})=\sum_{h=1}^{L}W_{h}^{2}\overset{ˆ}{V}({\overset{ˆ}{\mu }}_{Ph}),$$

where $W_{h}=\frac{N_{h}}{N}$.

By substituting the corresponding values of the third, fifth, fourth and sixth columns of Table 4 respectively in relations (18), (19), (20) and (21), the ratio and product estimation of the average number of cheating students of Shahid Chamran University of Ahvaz along with its variance estimation Based on the proposed randomized response method, 5.4977, 5.4594, 0.0726 and 0.0720 were obtained, respectively.

To check the conditions of the first and second propositions, since the parameters are unknown, their estimates are used. The required estimates are given in the seventh and eighth columns of Table 3. The conditions for establishing the first and second propositions according to these two columns are given in the ninth and tenth columns of Table 3. Based on these columns, in different colleges of the Shahid Chamran University of Ahvaz, ratio and product estimation of the average number of cheating students is preferable to the traditional method. Note that the notations related to the data analysis results are introduced in Table 2.

**Table 2:** Notations used to summarize the results of data analysis.

Col. No.	Notation	Description
i	h	Stratum number
ii	$s_{x}^{2}$	Auxiliary variable sample variance
iii	*s*_*xz*_	Sample covariance between the randomized response variable *Z* and the auxiliary variable *X*
iv	$\overset{¯}{x}$	Auxiliary variable sample mean
v	*μ*_*x*_	Population mean auxiliary variable
vi	${\overset{ˆ}{\rho }}_{xz}$	Estimation of the correlation coefficient between the randomized response variable *Z* and the auxiliary variable *X*
vii	$\frac{\left(p_{2}-p_{3}\right)}{2}\overset{ˆ}{R}\frac{s_{x}}{s_{z}}$	The expression required to check the conditions of Proposition 1, Proposition 2
viii	Yes or No	Yes (If the condition of Proposition 1 is met) or No (If the conditions of Proposition 1 are not met)
ix	Yes or No	Yes (If the condition of Proposition 2 is met) or No (If the conditions of Proposition 2 are not met)
x	${\overset{ˆ}{\mu }}_{Rh}$	Ratio estimation of the average number of cheating students
xi	${\overset{ˆ}{\mu }}_{Ph}$	Product estimation of the average number of cheating students
xii	$\overset{ˆ}{V}({\overset{ˆ}{\mu }}_{Rh})$	Ratio estimation of the variance of the average number of cheating students
xiii	$\overset{ˆ}{V}({\overset{ˆ}{\mu }}_{Ph})$	Product estimation of the variance of the average number of cheating students

**Table 3:** Summary of collected data.

College title∖Col. No.	i	ii	iii	iv	v	vi	vii	viii	ix
Literature and Humanities	1	4.5020	0.07309	14.8348	15.07	0.0120	−0.0335	Yes	Yes
Economics and Social Sciences	2	4.369	0.1967	14.990	15.37	0.033	−0.0344	Yes	Yes
Educational Sciences and Psychology	3	2.141	0.02911	16.473	15.92	0.007	−0.0211	Yes	Yes
Science	4	3.740	−0.0084	15.148	14.92	−0.002	−0.0296	Yes	Yes
Engineering	5	2.690	0.09523	14.692	14.78	0.021	−0.0288	Yes	Yes
Agriculture	6	2.531	−0.0907	14.441	14.38	−0.020	−0.0241	Yes	Yes

**Table 4:** The results of ratio and product estimation of the average number of cheating in the colleges of Shahid Chamran University of Ahvaz.

College title∖Col. No.	i	x	xi	xii	xiii
Literature and Humanities	1	5.5160	5.3452	0.1565	0.1560
Economics and Social Sciences	2	5.9470	5.6566	0.3567	0.3535
Educational Sciences and Psychology	3	4.9674	5.3185	1.0687	1.0675
Science	4	5.2202	5.3809	0.1637	0.1638
Engineering	5	5.6898	5.6223	0.3887	0.3868
Agriculture	6	4.9509	4.9930	0.4235	0.4251

## 7 Discussion and conclusion

In this article, a new quantitative three-stage randomized response method was presented and an estimator for the mean and variance of the sensitive variable was introduced. Using simulation, we have shown that the mean and variance estimators have acceptable performance. We presented ratio and product estimators of the mean of the sensitive variable using auxiliary variables. We proved that ratio and product estimators are better than the usual suggested estimators under certain conditions. By using stratified sampling method and ratio and product estimators, the average number of cheating in different colleges of Shahid Chamran University of Ahvaz in Iran in the first half of the academic year 2018-2019 was obtained. The estimate of the average number of cheating in the exams of Shahid Chamran University of Ahvaz was calculated by the ratio and product method as 5.4977 and 5.4594 respectively with a standard error of 0.0726 and 0.0720.

## CRediT authorship contribution statement

**Hamed Salemian:** Writing – original draft, Software, Resources, Project administration, Methodology, Investigation, Data curation, Conceptualization. **Eisa Mahmoudi:** Writing – review & editing, Writing – original draft, Supervision, Software, Project administration. **Osama Abdulaziz Alamri:** Writing – review & editing, Writing – original draft, Software, Methodology. **Javid Shabbir:** Writing – review & editing, Writing – original draft.

## Declaration of Competing Interest

The authors declare that they have no known competing financial interests or personal relationships that could have appeared to influence the work reported in this paper.

## Data Availability

Data will be made available on request.

## Appendix A Simulation codes

install.packages("ggplot2") library(ggplot2) runs <- 1000; sample_sizes <- c(50, 100,300, 500); mu_Y_values <- c(5,10,20); sigma_Y_values <- c(5, 10, 15); mu_S <- 0; sigma_S <- 1; b <- 9; p1 <- 0.25; p2 <- 0.25; p3 <- 0.5 results <- data.frame(SampleSize = mu_Y = sigma_Y = mu_hat = Abs_Bias_hat_mu_Y= V_hat = numeric(0)) for (mu_Y in mu_Y_values) { for (sigma_Y in sigma_Y_values) { for (n in sample_sizes) { mu_hat_values <- numeric(runs); V_hat_values <- numeric(runs); Bias_hat_values <- numeric(runs); Abs_Bias_hat_mu_Y <- numeric(runs) for (i in 1:runs) { S <- rnorm(n, mean = mu_S, sd = sigma_S); Y <- rnorm(n, mean = mu_Y, sd = sigma_Y) Z <- numeric(n) for (j in 1:n) { rand_val <- runif(1) if (rand_val <= p1) { Z[j] <- S[j] } else if (rand_val <= p1+p2) { Z[j] <- Y[j] } else { Z[j] <- b - Y[j] } } mu_hat_values[i] <- (mean(Z) - p1 * mu_S - p3 * b) / (p2 - p3) Bias_hat_values[i]<- mu_hat_values[i]-mu_Y Abs_Bias_hat_mu_Y[i]<- abs(mu_hat_values[i]-mu_Y) V_hat_values[i] <- var(Z) / (n * (p2 - p3)^2) } mu_hat_mean <- mean(mu_hat_values) Bias_hat_mean <-mean(Bias_hat_values) Abs_Bias_hat_mu_Y_mean <-mean(Abs_Bias_hat_mu_Y) V_hat_mean <- mean(V_hat_values) results <- rbind(results, data.frame(SampleSize = n, mu_Y = mu_Y, sigma_Y = sigma_Y, mu_hat = mu_hat_mean, Abs_Bias_hat_mu_Y=Abs_Bias_hat_mu_Y_mean,V_hat =V_hat_mean)) }}} print(results) ##################################################### mu_Y_values <- 5; sigma_Y_values <- 5; sample_sizes <- seq(50, 1000, by = 50) results <- data.frame(SampleSize = integer(), mu_hat = numeric(), Abs_Bias_hat_mu_Y = numeric(), V_hat = numeric()) for (n in sample_sizes) { mu_hat_mean <- numeric(); Abs_Bias_hat_mu_Y_mean<-numeric(); V_hat_mean <- numeric() for (i in 1:1000) { S <- rnorm(n); Y <- rnorm(n, mean = mu_Y_values, sd = sigma_Y_values); Z <- numeric(n) for (j in 1:n) { Z[j] <- sample(c(S[j], Y[j], 9 - Y[j]), size = 1, replace = TRUE, prob = c(0.25, 0.25, 0.5)) } mu_hat <- (mean(Z) - 0.25 * mean(S) - 0.5 * 9) / (0.25 - 0.5) Abs_Bias_hat_mu_Y<- abs(mu_hat - mu_Y_values) V_hat <- var(Z) / (n * (0.25 - 0.5)^2) mu_hat_mean <- c(mu_hat_mean, mu_hat) Abs_Bias_hat_mu_Y_mean<-c(Abs_Bias_hat_mu_Y_mean,Abs_Bias_hat_mu_Y) V_hat_mean <- c(V_hat_mean, V_hat) } results <- rbind(results, data.frame(SampleSize = n, mu_hat = mean(mu_hat_mean), Abs_Bias_hat_mu_Y = mean(Abs_Bias_hat_mu_Y_mean), V_hat = mean(V_hat_mean))) } plot_metric_vs_sample_size <- function(data, metric, metric_label) { ggplot(data, aes(x = SampleSize, y = get(metric))) + geom_point(size = 3) + geom_line(color="red") +labs(title = paste0(metric_label, " vs. Sample Size"), x = "Sample Size", y = metric_label) + theme_minimal() } p1 <- plot_metric_vs_sample_size(results, "Abs_Bias_hat_mu_Y", "Abs_Bias_mu_hat") p2 <- plot_metric_vs_sample_size(results, "V_hat", "V_hat_mean") print(p1) print(p2)
